# Epidemiology and Prognostic Significance of Rapid Response System Activation in Patients Undergoing Liver Transplantation

**DOI:** 10.3390/jcm10235680

**Published:** 2021-12-01

**Authors:** Marcus Robertson, Andy K. H. Lim, Ashley Bloom, William Chung, Andrew Tsoi, Elise Cannan, Ben Johnstone, Andrew Huynh, Tessa O’Halloran, Paul Gow, Peter Angus, Daryl Jones

**Affiliations:** 1 Liver Transplant Unit, Austin Hospital, Heidelberg, VIC 3084, Australia; bloomstaa@hotmail.com (A.B.); william.chung25@gmail.com (W.C.); andrewhtsoi@gmail.com (A.T.); cannanec@gmail.com (E.C.); ben.johnstone@austin.org.au (B.J.); ahuynh01@gmail.com (A.H.); tessa.ohalloran@gmail.com (T.O.); paul.gow@austin.org.au (P.G.); peter.angus@austin.org.au (P.A.); 2 Department of Medicine, University of Melbourne, Austin Hospital, Heidelberg, VIC 3084, Australia; 3 Department of Medicine, Monash University School of Clinical Sciences, Clayton, VIC 3168, Australia; andy.lim@monash.edu; 4 Intensive Care Department, Austin Hospital, Heidelberg, VIC 3084, Australia; daryl.jones@austin.org.au; 5 Department of Epidemiology and Preventive Medicine, Monash University, Melbourne, VIC 3004, Australia

**Keywords:** medical emergency team, rapid response team, rapid response system, liver transplantation, intensive care unit, cirrhosis

## Abstract

Patients undergoing liver transplantation have a high risk of perioperative clinical deterioration. The Rapid Response System is an intensive care unit-based approach for the early recognition and management of hospitalized patients identified as high-risk for clinical deterioration by a medical emergency team (MET). The etiology and prognostic significance of clinical deterioration events is poorly understood in liver transplant patients. We conducted a cohort study of 381 consecutive adult liver transplant recipients from a prospectively collected transplant database (2011–2017). Medical records identified patients who received MET activation pre- and post-transplantation. MET activation was recorded in 131 (34%) patients, with 266 MET activations in total. The commonest triggers for MET activation were tachypnea and hypotension pre-transplantation, and tachycardia post-transplantation. In multivariable analysis, female sex, increasing Model for End-Stage Liver Disease score and hepatorenal syndrome were independently associated with MET activation. The unplanned intensive care unit admission rate following MET activation was 24.1%. Inpatient mortality was 4.2% and did not differ by MET activation status; however, patients requiring MET activation had significantly longer intensive care unit and hospital length of stay and were more likely to require inpatient rehabilitation. In conclusion, liver transplant patients with perioperative complications requiring MET activation represent a high-risk group with increased morbidity and length of stay.

## 1. Introduction

Liver transplantation (LT) remains a life-saving intervention for patients with acute liver failure, end-stage liver disease and hepatocellular carcinoma. Recent studies indicate that LT candidates are becoming older, have a greater comorbidity burden, and increasing Model for End-stage Liver Disease (MELD) score, all of which increase the risk of perioperative clinical deterioration and complications [1,2]. Although LT is a routinely performed surgery with excellent outcomes, it is a major intraabdominal vascular procedure with significant hemodynamic and physiological stressors. Patients undergoing LT are commonly profoundly unwell, have a high risk for rapid clinical deterioration, and require significant hospital resources and intensive care unit (ICU) support [3]. Postoperative clinical deterioration is associated with mortality and poor graft survival [4,5].

The Rapid Response System (RRS) is a hospital-wide approach for the early recognition and management of inpatients identified as ‘high risk’ for acute clinical deterioration. The evolution of the RRS was prompted by evidence that deteriorating hospital patients commonly experienced ‘failure to rescue’ with subsequent development of serious adverse events, including death, cardiac arrest, and unplanned ICU admission [6]. The RRS has been introduced into hospitals worldwide aiming to identify, review and treat acutely deteriorating ward patients with a view to reducing serious adverse events [6,7]. As part of the RRS, a dedicated Medical Emergency Team (MET), Rapid Response Team or Critical Care Outreach team is activated in response to simple, objective, and reproducible criteria, to provide timely treatment and avoid a poor clinical outcome [8]. In Australia, the MET is usually an ICU-based clinical team [9].

The etiology of acute clinical deterioration events and their prognostic significance is poorly understood in LT patients. Only one previous study examined the frequency and characteristics of MET activation in LT patients. In a single-center retrospective study, 10% of LT patients required MET activation postoperatively, and these patients experienced an increased ICU and hospital length of stay, and higher in-hospital and one-year mortality compared to matched controls [10]. However, a significant number of LT candidates are hospitalized prior to LT but no previous study has investigated the significance of pre-LT MET activation in this cohort.

The objectives of this study were to (1) describe the epidemiology of MET activation in patients during the hospital admission in which transplantation occurred, (2) to determine the patient factors associated with MET activation, and (3) to assess the association between MET activation and patient outcomes, including mortality, unplanned admission to ICU, and length of stay.

## 2. Materials and Methods

### 2.1. Study Design, Setting and Population

We performed a cohort study of adult LT recipients (≥18 years) at the Victorian Liver Transplant Service at Austin Health (Victoria, Australia). Eligible patients were identified from a prospectively collected database and included consecutive recipients of a de novo or repeat LT over an 84-month period from 2011–2017. Patients receiving multi-visceral transplantation were excluded. The Victorian Liver Transplant Service accepts referrals for LT from the states of Victoria and Tasmania, covering a catchment of approximately 11.7 million people. The ICU has a 23-bed capacity which manages more than 2200 admissions annually. It operates a closed model where only ICU physicians can prescribe therapy. Following transplantation, patients were systematically admitted to the ICU. Upon discharge from ICU, patients are managed in a dedicated transplant ward staffed by transplant surgeons and physicians.

### 2.2. Medical Emergency Team (MET)

The MET was introduced at Austin Health in 2000 and is governed by the Department of Intensive Care Medicine. MET activations were designed to review medical emergencies and clinical deterioration except for cardiopulmonary arrest. A cardiopulmonary arrest was defined by loss of circulation or need for at least one of cardiopulmonary resuscitation, defibrillation, cardioversion, emergency pacing or emergency intubation, and this triggers activation of a Code Blue. The MET consists of an ICU registrar, internal (general) medicine registrar and ICU nurse equipped with materials and medications to recreate ICU care at the bedside within 5 to 10 min. The MET can be activated by any hospital staff member according to predefined physiological criteria (Table 1) and staff are mandated to activate the MET if a patient reached these criteria. Staff are also permitted to activate the MET if they are concerned about any other aspect of a patient’s clinical condition, referred to as the “staff concerned criterion”. This may include deterioration which has not reached thresholds based on vital signs, such as major bleeding, fall-related trauma, or uncontrolled pain. The MET at our institution reviews approximately 3500 patients annually, with postoperative patients comprising the largest group.

### 2.3. Study Outcomes and Variables

The primary outcome was MET activation, and the secondary outcomes were un-planned ICU admissions, length of stay, and mortality. We collected data on demographics, comorbidities, pre-LT location (home or hospital), length of stay, discharge destination, etiology of liver disease, MELD score at time of transplantation, liver disease complications (hepatic encephalopathy, ascites, variceal bleeding, hepatorenal syndrome, spontaneous bacterial peritonitis, sepsis), MET activation data (time of day, day of week, clinical trigger, vital signs, interventions performed during MET activation), and patient disposition (transferred to ICU, remained on ward, MET status reevaluated). Each MET activation was considered a separate event if a patient had >1 MET activation during admission. MET activations were further classified into those occurring pre-transplant (pre-LT MET) and post-transplant (post-LT MET).

Comorbidity burden was assessed using the age-adjusted Charlson Comorbidity Index (ACCI), a validated metric that predicts one-year mortality [11]. For the calculation of the ACCI, moderate to severe chronic kidney disease was defined as an estimated glomerular filtration rate < 60 mL/min/1.73 m^2^, current hemodialysis, previous kidney transplantation or hepatorenal syndrome managed with terlipressin; and severity of chronic liver disease was based on the Child-Pugh classification and MELD scores. Congestive cardiac failure was defined as ejection fraction of <40% on echocardiography or a documented clinical diagnosis of heart failure.

### 2.4. Data Source

The main data source was a prospective transplant database containing information on demographics, etiology of liver disease, MELD score, graft and donor characteristics, duration of LT surgery, and all pathology and radiology results. Two investigators independently reviewed medical records to identify MET activation and extract data. Discrepancies were arbitrated by an independent reviewer. A dedicated electronic MET reporting form is completed by the ICU team after each activation, which documents the indication for the MET activation, outcomes, and a provisional diagnosis of the medical condition responsible for the MET activation.

### 2.5. Statistical Analysis

We report the mean and standard deviation (SD) for normally distributed continuous variables, and the median and interquartile range (IQR) if distribution was significantly skewed. We used a χ2 test to measure the association between categorical data, and the Mann-Whitney test for continuous data with skewed distributions. Logistic regression was used to determine the association between baseline clinical variables (age, sex, MELD score, ACCI, cause of liver disease and several prespecified complications of chronic liver disease) and the primary outcome of MET activation. We used standard logistic regression as these clinical variables were time invariable in the context of this study, so patients experiencing >1 MET activation contributed only one unique data set. From univariable analysis, a modified stepwise approach was used to select variables into the multivariable model. Clinically meaningful variables with the largest odds ratios and a *p* < 0.10 were initially entered into the model, followed by variables with smaller odds ratios, while variables with *p* > 0.15 were removed. After all variables were considered, we retained variables with a *p* < 0.05. Models were compared using Akaike’s and Bayesian information criteria. Tests for interaction were conducted at a 1% level. We determined model calibration with the Hosmer-Lemeshow test. Model diagnostics included an assessment of the linearity of the logit of continuous variables, distribution of the model residuals, and the delta-beta method for influential cases. All analyses were performed using STATA 16.1 (StataCorp., College Station, TX, USA, 2020). A *p* < 0.05 was considered statistically significant.

## 3. Results

### 3.1. Patient Characteristics

There were 383 patients in the LT database. Two patients were excluded as multi-visceral transplant recipients. Characteristics of the remaining patients (*n* = 381) are summarized in Table 2. The median age was 56 years (65.4% male). Prior to LT, 257 of 381 (67.5%) patients were residing at home and presented to hospital solely for LT. The remaining 124 (32.6%) patients were already hospitalized with acute illness, of which 47 (12.3%) required ICU care pre-LT. The most common indications for LT were end-stage liver disease secondary to chronic viral hepatitis (hepatitis C, 33.2%; hepatitis B, 11.2%), hepatocellular carcinoma (36.7%) and alcohol-related liver disease (24.8%). The median MELD score at time of LT was 18 and the mean ACCI score was 5.0. Ascites was the commonest pre-LT end-stage liver disease-related complication, followed by hepatic encephalopathy and hepatorenal syndrome.

### 3.2. Tranplantation Characteristics

Most LT patients were recipients of donation after brain death, and only 27 of 381 (7.1%) patients were recipients of donation after cardiac death. Split LT was performed in 19 of 381 (5.0%) patients. There were no ABO-incompatible transplants performed during the study period. The mean cold ischemia time for the cohort was 6.4 h (SD, 1.9 h), and mean warm ischemia time was 0.8 h (SD, 0.2 h). The mean LT operation time was 8.0 h (SD, 1.9 h). All LT patients had intraoperative cell salvage and autologous red blood cell transfusions. The median intraoperative red blood cell transfusion volume in the database was 1.20 L (IQR, 0.76-2.06 L). All patients were routinely admitted to ICU postoperatively, where the median length of stay was 2.7 days (IQR, 1.6-4.8 days). None of these LT characteristics were associated with a statistically significant probability of post-LT MET activation (Table 2).

### 3.3. Medical Emergency Team Activation Characteristics

During the index admission, 266 MET activations were recorded in 130 of 381 (34.1%) patients (MET activation rate, 698 per 1000 LT admissions). Of these, 40% of MET activations occurred pre-LT and 60% occurred post-LT. Prior to LT, 106 MET activations occurred in 58 patients and post-LT, 160 occurred in 88 patients. Sixty-seven patients recorded a single MET activation, while 33, 10 and 20 patients recorded 2, 3 and >3 MET activations, respectively. Sixteen patients (4.2%) experienced a MET activation both pre- and post-LT. MET activation occurred a median of 11 days (IQR, 5-27 days) prior to LT in the pre-LT MET group and 11 days (IQR, 5–23 days) after LT in the post-LT MET group.

**Figure 1:**
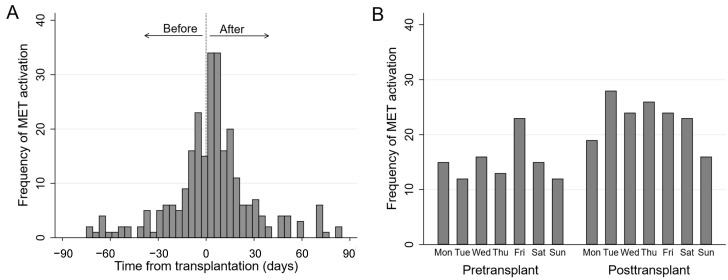
(**A**) The distribution of Medical Emergency Team (MET) activations relative to the day of transplantation (t = 0, vertical dashed line), showing that the peak occurrence of MET activation occurred in the first 7 days after liver transplantation. (**B**) The distribution of MET activation across the week in patients pre- and posttransplant.

The distribution of MET activations relative to the time of LT is shown in Figure 1. MET activations were mostly well distributed across the week, with a suggestion of greater MET activations on Friday pre-LT, but fewer MET activations on Sunday post-LT. MET activations were less frequent overnight (2300 to 0700 h) compared to other times but this difference was not statistically significant (*p* = 0.16). Further details of MET activation triggers, clinical observations and investigations are shown in Table 3. The most common triggers for MET activation were tachypnea (34.2%) and tachycardia (32.3%). Pre-LT, the common MET activation triggers were breathing disturbance (tachypnea and/or oxygen desaturation, 41.5%) and hypotension (24.5%); and post-LT, the common triggers were tachycardia (42.5%) and breathing disturbance (40.0%).

### 3.4. Factors Associated with Medical Emergency Team Activation

In relation to baseline characteristics (Table 2), patients transplanted for hepatocellular carcinoma were less likely to experience MET activation (22.3% vs. 44.2%, *p* < 0.001), with no other association found between etiology of liver disease and MET activation. Female patients were more likely to have MET activation (43.9% vs. 30.0%, *p* < 0.01). MELD score at LT was significantly higher in patients with pre-LT MET activations compared to those who did not receive a MET activation (27 vs. 16, *p* < 0.001) or received a post-LT MET activation (27 vs. 20, *p* < 0.001). The mean ACCI was lower in patients who experienced a MET activation (4.5 vs. 5.2, *p* < 0.001); however the absolute difference was small and of unclear clinical significance.

Preoperative complications of end-stage liver disease were also associated with a higher incidence of MET activation (Table 2). Patients with pre-LT MET activation were more likely to have experienced hepatic encephalopathy (65.5% vs. 38.7%, *p* < 0.001), ascites (79.3% vs. 57.8%, *p* = 0.003) and spontaneous bacterial peritonitis (34.5% vs. 20.6%, *p* = 0.039) compared to patients with no MET activations. Furthermore, patients who recorded a MET activation had a higher incidence of pre-LT sepsis (20.8% vs. 9.6%, *p* = 0.003) and hepatorenal syndrome (35.4% vs. 20.7%, *p* = 0.003), in both cases accounted for by a significantly higher incidence in the subset of patients receiving pre-LT MET activation (Table 2).

The results of the logistic regression analysis for patients experiencing at least 1 MET activation during the LT admission are summarized in Table 4. In the univariable analysis, female sex, a higher MELD score, hepatorenal syndrome and sepsis were associated with significantly higher odds of MET activation. Conversely, patients undergoing LT for hepatocellular carcinoma and patients with a higher ACCI had lower odds for MET activation.

In the multivariable analyses, several options were possible depending on whether the ACCI was considered. There was a moderately strong correlation between the ACCI and hepatocellular carcinoma (*r* = 0.53, *p* < 0.001) and, as such, both variables could not be modelled together possibly due to collinearity. In the selected model, on average there was a 79% higher odds of MET activation in female LT recipients, an 18% higher odds of MET activation for every 5 points increase in MELD score and a 68% higher odds of MET activation in the presence of hepatorenal syndrome. The model was a reasonable fit for the data (Hosmer-Lemeshow, *p* = 0.63) but the predictive value was modest, with an area under the receiver operating curve of 0.66.

Logistic regression was also performed separately for patients who were hospital inpatients > 24 h prior to LT (Supplementary Table S1) and for patients post-LT (Supplementary Table S2). In the pre-LT MET analysis, hepatic encephalopathy (adjusted OR 2.40, 95% CI: 1.11–5.17, *p =* 0.026), spontaneous bacterial peritonitis (adjusted OR 3.20, 95% CI: 1.30–7.89, *p =* 0.011) and greater pre-LT hospitalization days (adjusted OR 1.05, 95% CI: 1.02–1.08, *p =* 0.001) were independently associated with a higher odds of MET activation. Patients with a higher ACCI had lower odds of MET activation (adjusted OR 0.80, 95% CI: 0.65–0.99, *p =* 0.039). In the post-LT analysis, patients with a primary diagnosis of hepatocellular carcinoma had a lower odds for MET activation (crude OR 0.49, 95% CI: 0.29–0.84) but this was not independent of the ACCI or MELD scores (adjusted OR 0.68, 95% CI: 0.36–1.27).

### 3.5. Investigations and Management Initiated during MET Activation

Blood tests and diagnostic imaging were performed in 62.0% and 61.7% patients respectively (Table 3). In relation to interventions, intravenous fluid and blood product replacement were initiated in 38.0% and 13.5% patients, respectively. Antibiotics were commenced in 28.2%, and intubation or non-invasive ventilation was needed in 7.5% of MET activations. Infective complications were the most common precipitant for MET activation: 32% were related to sepsis, which was similar in both pre- and post-LT MET activations and 12.8% patients were diagnosed with a hospital-acquired pneumonia. In relation to the source of sepsis, patients experiencing a pre-LT MET activation were more likely to have enterococcal bacteremia (14.2% vs. 3.1%, *p =* 0.001) while post-LT MET activations had a higher incidence of hospital-acquired pneumonia (16.3% vs. 7.5%, *p =* 0.04). Bleeding complications were observed in 14.7% of MET activations and did not significantly differ between the groups.

### 3.6. Intensive Care Unit Admission

Following MET activation, 74.4% patients remained on the ward and 1.5% patients were taken directly to the operating theatre for emergency surgery and stabilization. The remaining 24.1% patients required an unplanned ICU admission, with 34.9% of pre-LT and 16.9% of post-LT MET activations resulting in unplanned ICU admission respectively. The median total post-LT ICU length of stay was significantly longer in patients who experienced any MET activation compared to patients who did not experience MET activation (126 h (IQR, 61–264 h) vs. 65 h (IQR, 39–115 h), *p* < 0.001) (Table 5).

### 3.7. Hospital Length of Stay, Mortality, and Discharge Destination

Table 5 summarizes the length of stay, mortality, and discharge destination. The median post-LT hospital length of stay was longer in patients who experienced MET activation compared to patients who did not experience MET activation (24 days (IQR, 15–40 days) vs. 13 days (IQR, 10–19 days); *p* < 0.001). No patient deaths were recorded during a MET activation. Overall inpatient mortality was 4.2%, with one additional death at 30-days post discharge. The inpatient and 12-month survival rates post-LT were 95.8% and 93.2% respectively. MET activation during the LT admission had no statistically significant effect on these survival rates. Patients who experienced MET activation were more likely to require inpatient rehabilitation rather than being discharged directly home, compared to patients without MET activation (45.2% vs. 15.2%, *p* < 0.001).

## 4. Discussion

We present the results of a large observational analysis of MET activations in patients undergoing LT and the association with clinical outcomes. In our cohort, 34% patients experienced at least 1 MET activation, most commonly due to respiratory disturbance. MET activation was associated with a 24% unplanned ICU admission rate, with the highest risk in the subset of patients experiencing a pre-LT MET activation. In addition, MET activations were associated with a prolonged post-LT ICU and hospital length of stay, but did not significantly affect inpatient or 30-day mortality.

This study is the largest to investigate the epidemiology and clinical significance of RRS activation in LT patients and the first to analyze MET activations in patients hospitalized prior to transplantation. Significant hospital resources are allocated to RRS to identify patients at high-risk of clinical deterioration with a view to improving outcomes and preventing adverse events. Despite its widespread integration into healthcare systems, there remains a paucity of literature regarding the utilization and efficacy of RRS in the solid organ transplant population. Indeed, only one previous study has investigated the epidemiology of MET activations in LT patients. Parmar et al. published a small single-center series of 174 LT recipients, of whom 18 experienced postoperative MET activation [10]. Like our study, MET activation was most commonly due to respiratory distress and associated with prolonged hospital and ICU length of stay, and a decreased likelihood of being discharged directly home.

One-third of patients in our cohort experienced clinical deterioration requiring MET activation during the hospital admission in which LT was performed. Importantly, this clinical deterioration could not be explained by an increased baseline comorbidity burden or older age, with a lower ACCI recorded in patients receiving MET activation. The MET activation rate of 698 per 1000 LT admissions in this study is significantly higher than other cohorts of medical or non-transplant surgical patients [12,13,14], and higher than the rate of 149 per 1000 LT admissions reported by Parmar et al. [10], although that study only included post-LT MET activations. Institutional and cultural factors leading to increased RRS utilization at the study site may partially account for this high MET activation rate [15], but it is important to note that MET utilization in LT patients is far higher than other surgical patients at our institution. For example, a recent analysis of patients undergoing major hip surgery demonstrated a 9% postoperative MET activation rate [14], even though these patients were, on average, 26 years older and had higher mean ACCI scores than LT recipients. This high rate of MET activation undoubtedly reflects the complexity of LT patients and a propensity for perioperative acute clinical deterioration. This observation also suggests that the MET are reviewing clinical deterioration in patients who may benefit from ICU-based treatments.

Consistent with other studies, the most common trigger for MET activation in our cohort was respiratory disturbance, manifesting as hypoxia or tachypnea. Postoperative pulmonary complications are well described post-LT [16,17,18,19,20], with at least one pulmonary complication noted in up to 59.1% patients [21]. Multiple risk factors for postoperative pulmonary complications have been identified, including older patient age, severity of liver dysfunction, greater number of perioperative blood products, postoperative duration of mechanical ventilation, and acute rejection during the hospital stay [17,18,21]. Postoperative pulmonary complications are recognized as a significant cause of morbidity, mortality, and increased hospital length of stay [17,19,20]. In our study, 40% of post-LT MET activations were secondary to respiratory disturbance, with 16.3% attributed to a hospital-acquired pneumonia. While this study was not designed to analyze whether MET activation could reduce the burden of postoperative pulmonary complications, it is interesting to note that, in contrast to other studies, MET activation for respiratory disturbance was not associated with higher mortality. Much less is known about the cause of clinical deterioration necessitating RRS activation in patients hospitalized pre-LT. We found that respiratory disturbance remained the most common cause of MET activation, but the diagnosis of hospital-acquired pneumonia was significantly less common at 7.5%.

Our study is the first to examine risk factors for clinical deterioration necessitating MET activation in LT patients during the hospital admission in which transplantation was performed. In our multivariable analysis, female sex, increasing MELD score and hepatorenal syndrome were independently associated with a higher risk of MET activation. The association between MELD score and patient outcomes is well described in LT patients [22,23]. Previous studies have demonstrated that patients with hepatorenal syndrome have higher post-LT mortality and hospital length of stay [24,25], but this is the first to establish hepatorenal syndrome as a risk factor for perioperative clinical deterioration. In relation to sex disparity in LT, fewer women than men received LT in our cohort, which is consistent with other studies [26,27]. It remains controversial whether there are sex differences in post-LT survival, with recent large studies finding no difference after adjusting for graft quality [28,29]. Multiple studies have shown women to be at higher risk of mortality or of becoming too unwell for transplantation while on the waitlist [26,27,30,31], and a single center study by Rubin et al. found that women on the transplant waitlist had higher levels of hospitalization than men [32]. The reason for these sex-specific vulnerabilities remains unclear. This is the first study to suggest that women have a higher risk of clinical deterioration in the perioperative period necessitating increased utilization of the RRS. Further studies are required to validate these findings.

### Study Strengths and Limitations

The Victorian Liver Transplant Unit currently performs more than 100 LTs annually. The spectrum of liver disease and comorbidities of this LT cohort improves the generalizability of our findings to other transplant centers with a RRS using similar MET activation criteria. We also utilized a prospectively collected database of LT patients, which improved the reliability of clinical data while avoiding missing data. By reporting the rate of MET activation and the association with increased ICU and hospital length of stay, we have highlighted the need for enhanced surveillance and monitoring of LT patients both pre- and post-LT and the development of more effective early warning systems to detect patient deterioration. To our knowledge, analysis of pre-LT MET activation has not been previously reported and our study demonstrates a high rate of clinical deterioration in this cohort.

This study has several limitations. Firstly, it is a single center study and MET activation data was retrospectively collected. Secondly, while the MET activation criteria at our institution are consistent with international guidelines, differences in activation criteria may limit generalizability. Rates of MET activation can also vary significantly between centers and are influenced by institutional factors and protocols. Thirdly, due to the study design, we could not exclude the possibility of missed MET activations (where a patient reached MET criteria without the MET being activated). However, previous hospital audits have demonstrated that a missed MET activation is rare (<5% of patients meeting MET criteria). Finally, as an observational study, it was not possible to establish causality in terms of the effects of the RRS activation on clinical outcomes.

## 5. Conclusions

LT patients with pre- or postoperative complications prompting MET activation represent a high-risk group with increased in-hospital complications, ICU, and hospital length of stay. Female patients, and patients with hepatorenal syndrome and higher MELD scores are at particularly high risk of clinical deterioration during the hospital admission in which LT is performed. Early identification and correction of factors which predispose to MET activation in this population may lead to improved outcomes and a reduction in healthcare costs.

## Figures and Tables

**Table 1:** Medical Emergency Team activation criteria.

Body System	Criteria
Airway	Obstructed airway Noisy breathing or stridor Problem with tracheostomy tube
Breathing	Any difficulty breathing Respiratory rate < 8/min Respiratory rate > 25/min Oxygen saturation < 90% despite oxygen
Circulation	Heart rate < 40/min Heart rate > 120/min Systolic blood pressure < 90 mm Hg Urine output < 50 mL over 4 h
Conscious state	Sudden change in conscious state Patient cannot be roused
Other	Staff member concerned Repeated or prolonged seizure Severe bleeding

**Table 2:** Baseline characteristics of patients by Medical Emergency Team activation status.

Characteristic	All Patients *n* = 381	No MET *n* = 251	All MET *n* = 130	Pre-LT MET *n* = 58	Post-LT MET *n* = 88
Age, median (IQR), years	56 (48–61)	57 (49–61)	55 (46–60)	55 (46–60)	55 (46–60)
Female sex, *n* (%)	132 (34.7)	75 (30.0)	57 (43.9) ^6^	28 (48.3) ^6^	37 (42.1) ^5^
MELD score, median (IQR)	18 (11–26)	16 (10–23)	21 (11–31)	27 (17–35) ^7^	20 (11–29)
ACCI, mean (SD)	5.0 (1.9)	5.2 (1.8)	4.5 (1.8) ^6^	4.4 (1.8) ^6^	4.5 (1.8) ^6^
**Liver disease etiology** ^1^					
Alcohol-related, *n* (%)	94 (24.7)	63 (25.1)	31 (23.9)	16 (27.6)	22 (25.0)
Chronic viral hepatitis, *n* (%) ^2^	163 (42.8)	110 (43.8)	53 (40.8)	22 (37.9)	38 (43.2)
Autoimmune liver disease, *n* (%) ^3^	85 (22.3)	55 (21.9)	30 (23.1)	17 (29.3)	16 (18.2)
Hepatocellular carcinoma, *n* (%)	140 (36.7)	111 (44.2)	29 (22.3) ^7^	7 (12.1) ^7^	22 (25.0) ^6^
Non-alcoholic steatohepatitis, *n* (%)	47 (12.3)	30 (12.0)	17 (13.1)	9 (15.5)	10 (11.4)
Other etiologies, *n* (%) ^4^	71 (18.6)	44 (17.5)	27 (20.8)	9 (15.2)	20 (22.7)
**Pretransplant location**					
Home resident, *n* (%)	258 (67.7)	196 (78.1)	61 (46.9) ^7^	0 (0) ^7^	56 (63.6) ^5^
Inpatient—hospital ward, *n* (%)	76 (19.9)	37 (14.7)	40 (30.8) ^7^	34 (58.6) ^7^	15 (17.0)
Inpatient—intensive care unit, *n* (%)	47 (12.3)	18 (7.2)	29 (22.3) ^7^	24 (41.4) ^7^	17 (19.3) ^6^
Pretransplant LOS, median (IQR), day	0.3 (0–7)	0.3 (0–0.6)	6 (0.3–19)	21 (10–29)	0.5 (0–7)
**Pretransplant complications** ^1^					
Hepatic encephalopathy, *n* (%)	154 (40.4)	97 (38.7)	57 (43.9)	38 (65.5) ^7^	32 (36.4)
Abdominal ascites, *n* (%)	231 (60.6)	145 (57.8)	86 (66.2)	46 (79.3) ^6^	55 (62.5)
Hepatorenal syndrome, *n* (%)	98 (25.7)	52 (20.7)	46 (35.4) ^6^	32 (55.2) ^7^	23 (26.1)
Spontaneous bacterial peritonitis, *n* (%)	69 (18.1)	43 (17.1)	26 (20.0)	20 (34.5) ^5^	12 (13.6)
Sepsis, *n* (%)	51 (13.4)	24 (9.6)	27 (20.8) ^6^	20 (34.5) ^7^	12 (13.6)
**Transplant characteristics**					
Donation after cardiac death, *n* (%)	27 (7.1)	20 (8.0)	7 (5.4)		6 (6.8)
Split donor liver graft, *n* (%)	19 (5.0)	8 (3.2)	11 (8.5) ^5^		6 (6.8)
Cold ischemia time, mean (SD), h	6.4 (1.9)	6.3 (2.0)	6.6 (1.8)		6.6 (1.6)
Warm ischemia time, mean (SD), h	0.8 (0.2)	0.8 (0.2)	0.8 (0.2)		0.8 (0.2)
Duration of surgery, mean (SD), h	8.0 (1.9)	7.9 (2.0)	8.2 (1.8)		8.4 (2.0)

^1^ Non-exclusive categories due to overlapping causes or features. ^2^ Three patients with hepatitis D coinfection. ^3^ Includes primary biliary cirrhosis, primary sclerosing cholangitis and autoimmune hepatitis. ^4^ Includes cryptogenic, drug-induced liver injury, polycystic liver, secondary sclerosing cholangitis, α1-antitrypsin deficiency, amyloidosis, Wilson’s disease, hemochromatosis, cystic fibrosis, and re-transplantation. ^5^ *p* < 0.05, ^6^ *p* < 0.01, ^7^ *p* < 0.001, compared to the no MET group. Abbreviations: MELD, Model for End-stage Liver Disease; ACCI, age-adjusted Charlson comorbidity index; LT, liver transplantation, MET, Medical Emergency Team; LOS, length of stay.

**Table 3:** Summary of Medical Emergency Team clinical data in liver transplantation.

Characteristic	All MET *n* = 266	Pre-LT MET *n* = 106	Post-LT MET *n* = 160
**Time of MET activation**			
0700–1459 h, *n* (%)	93 (35.0%)	36 (34.0%)	57 (35.6%)
1500–2259 h, *n* (%)	103 (38.7%)	44 (41.5%)	59 (36.9%)
2300–0659 h, *n* (%)	70 (26.3%)	26 (24.5%)	44 (27.5%)
**MET triggers** ^1^			
Oxygen desaturation, *n* (%)	28 (10.5)	15 (14.2)	13 (8.1)
Respiratory rate, *n* (%)	91 (34.2)	36 (34.0)	55 (34.4)
Tachycardia, *n* (%)	86 (32.3)	18 (17.0)	68 (42.5)
Hypotension, *n* (%)	47 (17.7)	26 (24.5)	21 (13.1)
Altered Glasgow Coma Scale, *n* (%)	31 (11.7)	17 (16.0)	14 (8.8)
Low urine output, *n* (%)	14 (5.3)	6 (5.7)	8 (5.0)
Clinical concern, *n* (%) ^2^	43 (16.1)	19 (17.9)	33 (20.6)
**MET observations**			
Heart rate, mean (SD), per min	108 (25)	100 (23)	113 (26)
Respiratory rate, mean (SD), per min	25 (8)	24 (7)	26 (8)
Systolic blood pressure, mean (SD), mmHg	115 (26)	107 (25)	121 (26)
Mean arterial pressure, mean (SD), mmHg ^3^	83 (17)	76 (16)	88 (17)
Mean arterial pressure <70 mmHg, *n* (%) ^3^	62 (23.9)	39 (37.9)	23 (14.5)
Temperature >38 °C	31 (6.0)	13 (12.3)	18 (11.3)
Glasgow Coma Scale score ≤13, *n* (%) ^4^	35 (13.3)	15 (14.3)	20 (12.5)
**Perioperative complications**			
Sepsis, *n* (%)	85 (32.0)	37 (34.9)	48 (30.0)
Pneumonia, *n* (%)	34 (12.8)	8 (7.5)	26 (16.3)
Enterococcus infection, *n* (%)	20 (7.5)	15 (14.2)	5 (3.1)
Staphylococcus infection, *n* (%)	2 (0.8)	1 (0.9)	1 (0.6)
Bleeding, *n* (%)	39 (14.7)	21 (19.8)	18 (11.3)
Venous thromboembolism, *n* (%)	3 (1.1)	2 (1.9)	1 (0.6)
**Interventions** ^1^			
Diagnostic imaging, *n* (%)	164 (61.7)	61 (57.5)	103 (64.4)
Blood tests, *n* (%)	165 (62.0)	67 (63.2)	93 (58.1)
Septic screen initiated, *n* (%)	100 (37.6)	39 (36.8)	61 (38.1)
Non-invasive ventilation/intubation, *n* (%)	20 (7.5)	7 (6.6)	13 (8.1)
Antibiotics, *n* (%)	75 (28.2)	33 (31.1)	42 (26.3)
Intravenous albumin, *n* (%)	72 (27.1)	45 (42.5)	28 (17.5)
Intravenous crystalloids, *n* (%)	29 (10.9)	7 (6.6)	22 (13.8)
Blood products, *n* (%)	36 (13.5)	25 (23.6)	12 (7.5)
Analgesia, *n* (%)	46 (17.3)	9 (8.5)	37 (23.1)
**Time at MET criteria before activation**			
<1 h, *n* (%)	79 (29.7)	26 (24.5)	53 (33.1)
1 to 4 h, *n* (%)	85 (32.0)	41 (38.7)	44 (27.5)
>4 h, *n* (%)	102 (38.3)	39 (36.8)	63 (39.4)

^1^ Categories were not mutually exclusive. ^2^ Includes fever > 38 °C, pain, and general concerns. ^3^ Missing diastolic blood pressure and mean blood pressure, pre-transplant *n* = 3, post-transplant *n* = 1; ^4^ Missing GCS score, pre-transplant *n* = 1. Abbreviations: MET, Medical Emergency Team; LT, liver transplantation.

**Table 4:** Logistic regression of Medical Emergency Team activation on clinical variables for the entire transplant admission.

Characteristic	Univariable		Multivariable	
	OR (95% C.I.)	*p*-Value	OR (95% C.I.)	*p*-Value
Age, per 10-year increase	0.87 (0.73–1.07)	0.212		
Female sex	1.83 (1.18–2.84)	0.007	1.79 (1.14–2.81)	0.011
MELD score, per 5-point increase	1.23 (1.10–1.38)	<0.001	1.18 (1.05–1.33)	0.006
Age-adjusted Charlson comorbidity index	0.80 (0.70-0.90)	<0.001		
Alcoholic liver disease	0.93 (0.57–1.53)	0.788		
Chronic hepatitis B or C virus infection	0.88 (0.57–1.36)	0.568		
Immunological disease ^1^	1.07 (0.64–1.77)	0.752		
Hepatocellular carcinoma	0.36 (0.22–0.59)	<0.001		
Non-alcoholic steatohepatitis	1.11 (0.59–2.09)	0.752		
Other cause of liver failure ^2^	1.23 (0.72–2.10)	0.442		
Hepatic encephalopathy	1.24 (0.81–1.90)	0.327		
Abdominal ascites	1.43 (0.92–2.22)	0.113		
Hepatorenal syndrome	2.10 (1.31–3.36)	0.002	1.68 (1.01–2.79)	0.044
Spontaneous bacterial peritonitis	1.21 (0.70–2.08)	0.491		
Other infection	2.48 (1.36–4.50)	0.003		

^1^ Includes primary biliary cirrhosis, primary sclerosing cholangitis and autoimmune hepatitis. ^2^ Includes cryptogenic, drug-induced liver injury, polycystic liver, secondary sclerosing cholangitis, α1-antitrypsin deficiency, amyloidosis, Wilson’s disease, hemochromatosis, cystic fibrosis, and re-transplantation.

**Table 5:** Summary of length of stay and patient survival outcomes after liver transplantation by Medical Emergency Team activation status.

Characteristic	All Patients *n* = 381	No MET *n* = 251	All MET *n* = 130	Pre-LT MET *n* = 58	Post-LT MET *n* = 88
Post-LT ICU LOS, median (IQR), h	78 (42–148)	65 (39–115)	126 (61–264) ^1^	137 (83–370) ^1^	124 (59–281) ^1^
Hospital LOS, median (IQR), days	18 (12–32)	14 (11–22)	31 (22–57) ^1^	49 (31–72) ^1^	29 (16–51) ^1^
Post-LT hospital LOS, median (IQR), days	15 (11–23)	13 (10–17)	22 (15–36) ^1^	24 (17–38) ^1^	23 (14–28) ^1^
Inpatient survival, *n* (%)	365 (95.8)	242 (96.4)	123 (94.6)	54 (93.1)	85 (96.6)
12-month survival, *n* (%)	356 (93.4)	235 (93.6)	121 (93.1)	53 (91.4)	83 (94.3)

^1^ *p* < 0.001 compared to the no MET group. Abbreviations: ICU, intensive care unit; LOS, length of stay; LT, liver transplantation; MET, Medical Emergency Team.

## Data Availability

The data that support the findings of this study are available on request from the corresponding author. The data are not publicly available due to privacy or ethical restrictions.

## Supplementary Materials

The following are available online at https://www.mdpi.com/article/10.3390/jcm10235680/s1, Table S1: Logistic regression of Medical Emergency Team activation in patients hospitalized prior to liver transplantation; Table S2: Logistic regression of Medical Emergency Team activation post liver transplantation.
