# Supplementary material for: Epidemiology and Prognostic Significance of Rapid Response System Activation in Patients Undergoing Liver Transplantation
Source: J Clin Med. 2021 Dec 1;10(23):5680. doi: 10.3390/jcm10235680 (PMC8658097; doi:10.3390/jcm10235680)
Supplement: Supplementary file 1 [file jcm-10-05680-s001.zip › Supplementary Table S1.pdf]

Journal of
Clinical Medicine

MDPI

Table S1. Logistic regression of Medical Emergency Team activation in patients hospitalized prior to liver transplantation (*n*=138)

Characteristic	Univariable		Multivariable	
	OR (95% C.I.)	*p*-value	OR (95% C.I.)	*p*-value
Age, per 10-year increase	0.95 (0.70-1.29)	0.75		
Female sex	1.20 (0.61-2.36)	0.60		
MELD score, per 5-point increase	1.11 (0.94-1.29)	0.21		
ACCI index, per point increase	0.85 (0.71-1.02)	0.09	0.80 (0.65-0.99)	0.039
Pre-LT LOS, per day increase	1.05 (1.02-1.08)	0.001	1.05 (1.02-1.08)	0.001
Alcoholic liver disease	1.80 (0.79-4.06)	0.16		
Chronic hepatitis virus infection	1.72 (0.83-3.55)	0.15		
Immunological disease	1.16 (0.55-2.47)	0.69		
Hepatocellular carcinoma	0.71 (0.26-1.90)	0.49		
Non-alcoholic steatohepatitis	1.45 (0.54-3.91)	0.46		
Other cause of liver failure	0.43 (0.18-1.01)	0.053		
Hepatic encephalopathy	2.44 (1.22-4.91)	0.012	2.40 (1.11-5.17)	0.026
Abdominal ascites	2.43 (1.11-5.28)	0.026		
Hepatorenal syndrome	1.43 (0.73-2.82)	0.30		
Spontaneous bacterial peritonitis	2.98 (1.32-6.76)	0.009	3.20 (1.30-7.89)	0.011
Other infection	1.81 (0.85-3.85)	0.12		

Note: Patients with a pre-transplant length of stay <24 hr (*n*=243) were excluded as the risk of MET activation was negligible.
Abbreviations: MELD, Model for end-stage liver disease; ACCI, Age-adjusted Charlson comorbidity index; LT, liver transplantation; MET, Medical Emergency Team; LOS, length of stay.
