# Supplementary material for: Epidemiology and Prognostic Significance of Rapid Response System Activation in Patients Undergoing Liver Transplantation
Source: J Clin Med. 2021 Dec 1;10(23):5680. doi: 10.3390/jcm10235680 (PMC8658097; doi:10.3390/jcm10235680)
Supplement: Supplementary file 1 [file jcm-10-05680-s001.zip › Supplementary Table S2.pdf]

Journal of
Clinical Medicine

MDPI

Table S2. Logistic regression of Medical Emergency Team activation post liver transplantation (n=381)

Characteristic	Univariable		Multivariable	
	OR (95% C.I.)	*p*-value	OR (95% C.I.)	*p*-value
Age, per 10-year increase	0.87 (0.70-1.07)	0.18		
Female sex	1.51 (0.93-2.47)	0.10		
MELD score, per 5-point increase	1.11 (0.98-1.25)	0.10	1.05 (0.93-1.20)	0.43
ACCI index, per point increase	0.84 (0.73-0.96)	0.010	0.89 (0.76-1.04)	0.15
Pre-LT LOS, per day increase	1.00 (0.98-1.02)	0.95		
Alcoholic liver disease	1.02 (0.59-1.78)	0.94		
Chronic hepatitis virus infection	1.02 (0.63-1.65)	0.93		
Immunological disease	0.72 (0.40-1.32)	0.29		
Hepatocellular carcinoma	0.49 (0.29-0.84)	0.010	0.68 (0.36-1.27)	0.22
Non-alcoholic steatohepatitis	0.89 (0.42-1.86)	0.75		
Other cause of liver failure	1.40 (0.78-2.50)	0.26		
Hepatic encephalopathy	0.80 (0.49-1.31)	0.38		
Abdominal ascites	1.11 (0.68-1.81)	0.68		
Hepatorenal syndrome	1.03 (0.60-1.77)	0.92		
Spontaneous bacterial peritonitis	0.65 (0.33-1.28)	0.22		
Other infection	1.03 (0.51-2.06)	0.94		
Any pre-LT MET activation	1.33 (0.71-2.50)	0.38		
Donation after cardiac death	0.95 (0.37-2.43)	0.91		
Split liver allograft recipient	1.58 (0.59-4.28)	0.37		
Warm ischaemia time, per hour	1.18 (0.30-4.71)	0.81		
Cold ischaemia time, per hour	1.07 (0.95-1.20)	0.29		
Total ischaemia time, per hour	1.06 (0.95-1.19)	0.29		
Surgical duration, per hour	1.04 (0.92-1.18)	0.50		

Abbreviations: MELD, Model for end-stage liver disease; ACCI, Age-adjusted Charlson comorbidity index; LT, liver transplantation; MET, Medical Emergency Team; LOS, length of stay.
